# The Prevention of Disease—A Problem for All Physicians1Read by invitation before the New York Academy of Medicine, February 1, 1894. Published in the *New York Medical Journal*, April 14, 1894.

**Published:** 1894-06

**Authors:** William Warren Potter

**Affiliations:** 284 Franklin Street; Buffalo, N. Y.


### THE PREVENTION OF DISEASE.—A PROBLEM FOR ALL PHYSICIANS.[1]

By WILLIAM WARREN POTTER, M. D., Buffalo, N. Y.

### INTRODUCTION.

The highest office of the physician of modern times is and ever must be in the exercise of his functions toward the prevention of disease. This is neither new nor original, but is, nevertheless, as true as when it was first uttered in reference to modern physicians and modern medicine.

In a medical society meeting it is far more entertaining and even fascinating to describe a brilliant clinical success, either in surgery or medicine, than it is to discourse upon the methods of prevention as applied to the maladies of mankind. But it would be unseemly to address this Academy in general session assembled, composed as it is of men who are distinguished in all the specialties of their art, upon any one branch thereof expecting to attract either the ear or the thought of anything like a considerable number, to say nothing of a majority. But every one is or ought to be interested in the prevention of disease. Moreover, the time has fully come for the discussion in all medical societies by all medical men of the manifold questions relating to preventive medicine.

The problems that are involved in this department of medicine must interest the general practitioner, whether of medicine or surgery; likewise all specialists in the several departments and branches of medical science and art; nor can the laboratory teacher, whether he be chemist, pathologist, physiologist or bacteriologist, escape the necessity of a knowledge of how to prevent the diseases whose infection and contagion he is absorbed in studying and analyzing. What man is there who bears the honorable title of "Doctor of Medicine," no matter what his specialty in teaching or practice, who escapes the sometimes intelligent and always persistent interrogations of his patients or friends with reference to the best methods of preventing the spread of cholera, smallpox, diphtheria, typhoid fever and the like? He must be prepared to offer an intelligent opinion on all such questions, as well as on those pertaining to plumbing, sewerage, drainage, ventilation and every detail of house sanitation. Ignorance on, or indifferent acquaintance with, these subjects is a confession of

---

1. Read by invitation before the New York Academy of Medicine, February 1, 1894. Published in the *New York Medical Journal*, April 14, 1894.

unfitness to retain his title. Happily, however, some of the exanthematous fevers have not now the same dread to humanity as formerly, since the triumph of medicine in solving the methods of their prevention and cure. But if this be true in a certain degree, there is being added to the list a number of maladies that formerly were not thought to be contagious or at least infectious, but have now become well known to be so through the investigations and studies of clinicians and men experienced in the science of bacteriology.

Permit me to illustrate by accentuating the importance of a better knowledge concerning the prevention of the propagation of a few of the more serious diseases of this class.

### THE PREVENTION OF CONSUMPTION.

I may refer first to one disease—namely, consumption, that comparatively lately has become known to the profession as an infectious disorder—a malady that is destroying more inhabitants of the globe than almost any other known disease, and that last year proved fatal to more than 6,000 residents of your own city.[1]

While it is true that much has been done of late to control its spread and to abridge its ravages in localities, yet how many physicians are ready to admit even now that it is an infectious malady, and that it may be conveyed to a healthy person who is habitually in the atmosphere of a consumptive? If it were possible to apply the knowledge which a few of the more advanced—a small minority—of our profession now possess with reference to the propagation of consumption and to disseminate this knowledge amongst the mass of physicians, it would be serving humanity in the highest possible way to concentrate the efforts of the entire force toward preventing the spread of this direful disease. In order to do this effectively, it would become necessary at the outset to establish an absolute inspection of all foods of an animal nature, as well as supervision of the care of all animals that furnish food for mankind, with a view to a rigid condemnation and destruction of such as prove unfit.

Again, if we should undertake to prevent the spread of consumption by the application of all the intelligent means at our command, it would further involve imperious supervision over the food that our cows eat and their stable care, as well as a like supervision over the animals that are slaughtered for our daily

1. Report of Dr. Hermann M. Biggs to the Health Department of New York City.

consumption. It also further involves the inspection, by a competent veterinary surgeon, of all cows that afford milk supply to our cities and villages and of all the animals that are slaughtered for purposes of food, to the end that every tuberculous beast shall be killed and its body cremated. I consider it essential that the latter order shall be enforced with great exactitude. If any portion of a tuberculous animal, excepting its hide, horns, and hoofs, be permitted to enter the avenues of commerce, there is danger of the propagation of disease, and I know of no way to effectively abolish such danger excepting to imperiously insist upon the incineration of all tuberculous carcasses.

But we must not stop here; our food supervision must extend to all domestic birds, fowls, and animals whose milk or flesh we use for food. The quantities of tuberculous food consumed by the people assuredly are enormous, and it will require the most rigid system of inspection and supervision, by most skilled, honest and well-paid officials, to interrupt, cut short or prevent traffic in these dangerous and unwholesome aliments.

Besides this, the circumspection and control of railway and steamboat travel, as well as hotel life, would become necessary, and this would simply arouse an opposition from every traffic official and landlord in the country, to say nothing of the indignation of the travelers themselves. Yet none such would complain of the exercise of an absolute authority with reference to restraining the transit of smallpox patients, or those affected with diphtheria or cholera. Nevertheless, these maladies, one and all, have not at present one scintilla of the importance connected with their destructive powers that has consumption.

Another most important step toward the prevention of the propagation of consumption consists in the prohibition of the intermarriage of tuberculous subjects. If a man has tubercular disease he should not be allowed to marry a healthy woman, and thus subject her to the danger of contracting the malady from cohabitation with her consort, to say nothing of the liability of propagating tuberculous offspring. For like reasons, a tuberculous woman should be prohibited from marrying a healthy man. But, if there are cogent reasons why these should not intermarry, there are also forceful arguments that may be offered against the intermarriage of two tuberculous individuals, that is to say, a man and a woman who either have tuberculous disease or who have come from tuberculous families. I make this assertion at random without desiring at this time to enter into a discussion as to whether the laws of heredity govern in consumption or not. But of this I am certain, that for a man with existing tubercular disease to marry a woman of tubercular parentage would be tempting fate with a rude audacity deserving a better cause. The offspring of such a marriage, to say the least, would offer a favorable soil for the growth of the tubercle bacillus. I earnestly hope that before long an enlightened legislature will enact statutes that will prohibit such marriages.

Still another step in the direction of prevention consists in the establishment of separate hospitals for consumptives and the adoption of measures looking toward their isolation. But here most bitter opposition will be encountered from those who are governed by sympathy, affection and kinship. Nevertheless, it were well-nigh impossible to prevent the spread of this fateful malady, unless the methods and habits of life of its victims assuredly can be controlled. It is admitted by those most familiar with the methods of the propagation of the disease that the sputum of the consumptive constitutes a focus of special danger. Hence, to deal intelligently with the question of prevention, the sputum must be sterilized as soon as it is thrown off. Nor will it answer to deal with it in a half-hearted, indifferent or slipshod manner; the sterilization must be absolute and immediate, and this can only be accomplished under the eye of a trained expert who knows no law but that of perfect cleanliness and absolute sterilization, and who will not be diverted from the end in view by fear, favor or affection. I know of no way to effectively enforce rules to prevent the propagation of consumption, except to remove tuberculous persons from all possibility of contact, direct and indirect, with the healthy community.

Without doubt, independently of the infection by food, the most serious menace comes from the respiration of infected dust, and it needs no specious ratiocination to prove to such an audience as this, that the one great means of infecting dust is through the sputum of consumptive patients. It has recently been said by a writer in the *British Medical Journal* that it would be well for the medical profession at large to recognize the infectiousness of tuberculous sputum and the danger of letting it dry into dust. Marpman (*Centralblatt für Bacteriologie und Parasitenkunde*, Band XLV. 8, pp. 228-234, *Med. Chron.*, November, 1893,) examined the dust of a frequented street in Leipzig and found the bacillary remains on about 80 per cent. of samples of dust examined. From the researches of Marpman we are taught that street dust may easily prove a source of infection, and that people should be taught to desist from expectorating in thoroughfares or public places. Houses that have been occupied by consumptives, too, should receive the most thorough renovation before further use.

It cannot be denied that already much has been done to limit the spread of tuberculosis, as a careful examination of statistics would prove, but these we have no time here and now to enter into. The results already accomplished have been outside of the prevention of intermarriage, inspection of food, attempt at isolation, or sterilization of sputum, and have been mainly achieved through the adoption of improved sanitary regulations—better nutrition, purer air and other influences of like character.

I stand here to plead for the enforcement, in addition to these well-known sanitary measures, of the prevention of intermarriage, of the rigid inspection of animals that furnish food and the isolation of tuberculous patients, and I ask this Academy to stand together as one man until it has brought about such change in public sentiment and wrought such improvements in statutory law as shall make all these conditions possible. I believe it will be yet just as possible to quarantine and control the methods and habits of life of the consumptive as it is now those of a smallpox, yellow fever or cholera patient. At all events, I certainly hope that an enlightenment of the people on this subject will lead to such results.

### TYPHOID FEVER.

Turning from the consideration of tubercular disease we approach another infectious malady which has attracted the attention of physicians and sanitarians for more than a generation. In July, 1845, Dr. Austin Flint, Sr., then of Buffalo, and afterward one of your distinguished academicians, published in the *American Journal of Medical Sciences* an account of an epidemic fever which occurred at North Boston, Erie Co., N. Y., during the months of October and November, 1843.

It appears that in September, 1843, a young man from Massachusetts stopped at Fuller's tavern in North Boston. He had been unwell for several days, a diarrhea being among the most prominent and early of his symptoms; he was first seen by a physician only six days before his death, who found him suffering from diarrhea, low muttering delirium, sordes and other symptoms that grouped we nowadays characterize as a typhoid condition. He lived twenty-eight days, and from this case the fever spread to other members of Mr. Fuller's family and to those living nearest to his house. Seven of Mr. Fuller's family had the fever, of whom three died. Twenty-one other cases occurred in five families, all being within ten rods of Mr. Fuller's house, of whom seven died, making in all, including the stranger, twenty-eight cases and ten deaths. The whole population of the hamlet amounted to forty-three persons; none were attacked who were over twenty-three years old, the youngest was only one year old and a large proportion were children.

Dr. Flint visited the locality of the epidemic, during which time he made a post-mortem examination of the body of a child about twelve years old, and took notes at the bedside of the histories of nine cases together with the symptoms presented at the time of his visits. It was his first distinguished success as an investigator. He found and diagnosticated a fatal disease then unknown in Western New York, traced it from New England to the hamlet of North Boston, distinctly established its contagion and found its focus in a particular well of water which had been poisoned by the excreta of an original case. Further use of this well was prohibited. The cases then existing recovered and no new ones appeared. The work was done with all the precision and completeness that characterizes the very best detective work and with all the skill and acumen of the scientific clinician. Nothing was omitted that would contribute to the completeness and convincing power of the evidence adduced, and the published report has become a classic in this country and in Europe. It also formed the basis of a series of essays that Dr. Flint subsequently wrote on typhoid fever, which were published in this journal.

Thus was established for the first time a direct relationship between poisoned excreta and the contagion of typhoid fever. I wish we also could say that there had been established an absolute knowledge of the contagium of this destructive malady and the methods of its implantation. Who can say today with absolute certainty that the infection of typhoid can be prevented in a given case? Who has yet been able to solve the subtlety with which the bacillus of typhoid fastens itself upon the organism, or who can say what is the most favorable soil for its implantation? Medical societies are constantly debating these questions and the pages of medical journals are teeming with learned expositions of the various and manifold minutiæ of the subject. I listen to these debates and am to a certain extent familiar with journalistic literature. Yet I fail to find a satisfactory answer to the various questions bearing on the communicability of typhoid or any final and determined method of preventing its propagation. There are nearly as many opinions on these several details as there are participants in the debates or writers in periodicals. All this indicates that there is much yet to be done in experimental research, clinical observation and bacteriological study in relation to typhoid fever. If a conclusion is ever to be reached thereon that will be accepted as final by the profession, it must be through the combined efforts of clinicians, pathologists, bacteriologists and sanitarians. In the city of Chicago alone, from January 1, 1890, to November 1, 1893, there have been, according to published reports,[1] 5,087 deaths from typhoid fever, an average of 110 a month. This indicates that during this period there have been between thirty and forty thousand cases. There are, says Vaughan, annually 50,000 deaths in this country from typhoid fever, and half a million sick with it.[2] If it were possible to estimate the money lost to the community in salaries, wages and expenses from this one disease, the sum would be something enormous to contemplate, and yet there is a great outcry in most cities against the ordinary expenses of the health department. This embarrasses the officials, and the evil should be corrected through an enlightenment of the masses on the subject. If their sympathies cannot be reached from the humanitarian side of the question, then their interests must be appealed to from its economic aspects.

### DIPHTHERIA.

Similar statements may be offered with reference to diphtheria. Who can say what is the precise method of the propagation of this subtle poison? Who has yet ventured to tell us absolutely how it enters the system, what is its favorite soil, and why under apparently like conditions one individual contracts the disease, while another has only a mild malaise, and still another goes scot free? Who has told us with absolute certainty what part mephitic vapors play in the causation of this disease? In nearly all the large cities of the world during every month of the year, cases of diphtheria are reported ranging all the way from a few scattering victims to many hundreds and even thousands, with a fearful

1. *Medical Record*, December 30, 1893.
2. *Medical News*.

percentage of mortality. Here is another field where moot points are many, opinions widely differ and satisfactory conclusions are still in the air. Shall it be said during all the future years that diphtheria baffles all attempts at prevention? That it paralyzes the efforts of the medical profession to control it or to fix its habitat? This would be a sad confession of weakness and a stigma upon the science of medicine that ought not to be permitted to remain. Let us address ourselves to the study of its prevention with an assiduity that will at least deserve success.

### GONORRHEAL INFECTION IN WOMEN.

But one other example, yet in a most important field, will be cited at this time. I refer to gonorrheal infection in women. It is doubtful if any disease is capable of propagating more of sickness, woe and misery than a neglected or uncured gonorrhea. In view of recent researches, and especially of clinical experiences evolved at the operating table, it is now admitted that what was taught and written relating to the treatment of this infection prior to 1872 was little better than so much chaff; and this remark applies with equally cogent force to the acute stage in the male as to the chronic stage in the female. Though it is true that the medical profession gave little heed to the notes of warning sounded by Noeggerath when he published his opuscule in 1872, now, after twenty years of continual hammering, the foremost men in medicine have finally become aroused to the dangers that lurk in an uncured or latent gonorrhea. This is especially true of genito-urinary surgeons and of those gynecologists who practise pelvic surgery. Other specialists and clinicians who listen to their debates and read such convincing expositions of the subject as have been given on the one hand, for example, by your distinguished academician, Brewer, and on the other hand by Cushing, of San Francisco, have accepted in a measure their teachings.

Brewer has conclusively demonstrated in a clinical history that after six years had elapsed since infection, and where upon examination no secretion could be pressed from the urethra, yet endoscopic examination revealed granular patches and congested areas in the neighborhood of the bulb and behind a stricture of large caliber, measuring 33°. In this case the microscope showed several characteristic colonies of gonococci. Six weeks afterward, contrary to advice, this man married, and two weeks later had communicated the infection to his wife, in whom it went throught he various stages of inflammation and resulted in abscess.[1]

Cushing has shown that a woman who had been infected five years previously, without intermediate exposure, finally married and infected her husband who had a severe urethritis which resulted in a perineal abscess. Cushing removed both tubes and ovaries that were distended with pus in which gonococci were found. The secretion from the urethra of the husband was examined and gonococci demonstrated without difficulty. Here, then, are two cases—one in which the infection was carried by the male for six years and then communicated to his newly wedded wife; and the other in which it was carried by the patient for five years and then communicated to her husband, who had all the symptoms of acute gonorrhea resulting in abscess.[2]

I have cited these two examples as types of this disease showing its subtlety, how it lurks in some nook or cranny of the genital tract for years, thence to be conveyed to an innocent spouse or consort in whom it lights up a disease that ravages the entire pelvic cavity. The list might be prolonged indefinitely, but this is neither the time nor place to recite lengthy clinical records.

Only within the last few years have physicians been ready to admit the terrible destructiveness of this malady. Until lately most physicians or surgeons who have been called upon to treat the disease in its acute stages, whether in the male or female, have generally regarded it in a light or trifling manner, contenting themselves for the most part with ministering to the first distress, but paying little heed to its secondary probabilities. The victims of this disease, too, have been taught to regard it as a mere trifle that a few urethral or vaginal injections and a few doses of copaiba assuredly would cure, without danger of any secondary complications, and that even a long standing gleet had little or no significance beyond the mere personal discomfort that it caused. Only the few even consulted physicians, while the many borrowed a syringe and a prescription from a friend or obtained the same from a kindly and sympathizing druggist who, for a few shekels, is ever ready to assure the sufferer that his disease is of slight consequence, that he will be well of it in a few days, and that all he

1. The Contagiousness of Chronic Urethral Discharges. *Jour. of Cutaneous and Genito-Urinary Dis.*, March, 1891.

2. Contribution to the Study of Pelvic Disease. *Trans. Am. Ass'n of Obst. and Gyn.*, Vol. I., 1888, p. 17.

needs is a few injections with "No. 1" and a few days' dosing with "No. 2."

The literature of this disease is being almost entirely rewritten, and the teachings of the schools are recasting in view of the awful consequences of neglect to cure or prevent the propagation of this subtle, deceptive and virulent infection when once it has been contracted. It has come to pass that a woman who accepts an offer of marriage finds herself treading upon dangerous ground—a danger far more serious than those contemplated by mother Eve when she entailed her sex—and it becomes a problem of the most serious import when her answer may fetch her a spring of woes unnumbered. I speak seriously, for it is a subject that demands our most thoughtful consideration. If we would stamp out the frightful malady, or even prevent its propagation to innocent persons, we must, as a profession, exert ourselves to the utmost to bring enlightenment to the people and to enforce an intelligent application of the laws of prevention. We have been taught to regard syphilis as the most destructive of the venereal diseases, but I believe gonorrhea to be a far more serious disease than syphilis, and that it plays more dreadful havoc with community. We are seeing constantly fewer and fewer syphilitics, while gonorrheics are increasing in number. Fortunately, though, the secondary consequences of the disease are becoming better understood. I am well aware that difficulties surround this subject almost greater than any other, owing to its extreme delicacy ; nevertheless, the fulness of time has arrived when action must be taken to arrest a disease that leaves such horrible consequences in its track, and I appeal to this Academy to formulate and carry out some plan that shall have for its purpose the enlightenment of the community, the education of physicians, and the enactment of such statutes as shall prevent others than physicians from prescribing or in any manner giving advice as to the treatment of gonorrhea.

### CONCLUSIONS.

In my early youth I learned to look to the New York Academy of Medicine as the leader of medical thought in this country. My father, a physician, so taught me, as a child receives impressions from the horn-book on his mother's knee. It is here that medical men from all quarters of the globe are received and entertained at your meetings and go away with new inspirations, renewed energy and increased intelligence. This academy becomes, perforce, a mighty instrument for the advancement of medical science and improved methods in medical art. One of the great orators of this metropolis asseverated in his speech at Chicago on Manhattan day that the World's Fair had taught us that art is more god-like than science, for while science discovers, art creates. I believe this applies as well to medicine as to other arts and sciences. I ask you to remember that I am not unmindful of the great work this academy has inaugurated from time to time during its history with reference to the prevention of disease, nor of the fact that it is still laboring with might in that field. When this academy speaks, the world moves. When she asks that certain methods and certain means be adopted with reference to improvements in quarantine, they are done. When she recommends the board of health to adopt certain measures with reference to the details of preventive medicine, the board at once accepts its conclusions and adopts them. It has gratified me to observe very recently the splendid action taken by the academy with reference to the creation of a National Bureau of Health, and I sincerely hope its recommendations may be given form and force in the immediate future through national statutory law.

But it is not as sanitarians, or public health officers, or as administrators of state medicine, that I now appeal to this academy of medicine. I beseech you, each and every one, in your capacities as individual members, to do your parts toward inaugurating a fashion, that shall have for its purpose discussions relating to the prevention of disease at the general meetings of this academy to the exclusion of all other scientific work. The report of these proceedings given to the medical journals and published as *ex cathedra* edicts will arrest the attention of physicians all over the world, and thus serve to begin a campaign of education on the several questions to which I have called attention, as well as many others that for obvious reasons could not be specified on this occasion. I have merely attempted to accentuate those that seemed to me of greatest importance. Cholera, smallpox and even yellow fever are no longer regarded as plagues of humanity, but consumption, diphtheria, typhoid fever and gonorrhea are slaying or invaliding *unnecessarily* hundreds of thousands annually.

The laity must be educated to regard consumption as a communicable disease, and to accept laws and regulations calculated to restrict its propagation and restrain its victims within the limitation of safety so far as uninfected individuals are concerned. But rules, too, must be formulated for the prevention of the spread of diphtheria and typhoid, and these must be insisted upon without deviation, and the community must learn to yield a hearty support to such beneficent and humane methods as have for their sole and distinct object the protection of the individual. Finally, I hope that this academy will address itself seriously to the eradication, prevention and cure of that loathsome, subtle and destructive disease, gonorrhea, that is directly and indirectly bringing to invalidism or death so many innocent thousands throughout the civilized world. I am well aware that all these questions are as well known to you as to me—perhaps even better; I am only attempting to recall them to your minds for the purpose of stimulating or encouraging renewed energy in their consideration.

Pardon me, Mr. President, if I have said more than I ought to have said, or more than there was occasion to say. I have spoken from a deep conviction of the importance of the subject, and with a realizing sense that I am standing in the presence of men who are doing as much as any other group of equal numbers toward the prevention of the diseases in question, as well as others that have not been mentioned. I know, too, that your sections are made up of recognized men of exceptional skill in their several departments, and whose work goes out every month to the professional world in brilliant and well-recorded achievements. Your orthopedists are restoring hundreds of maimed children to usefulness and self-support; your ophthalmologists are restoring to sight thousands of eyes in every year, and are preventing the beginning of destructive processes in many thousands more; your gynecologists are restoring to health numberless invalid women—wives, mothers and sisters—who would otherwise drag through life a miserable existence or obtain all too early sweet relief in death; and so on through the entire list of your splendidly organized sections. But, in spite of all this, let me implore you to set apart at least one meeting in every month, if not both, where all the splendid talent of these sections shall merge itself for the discussion of problems relating to the prevention of disease; and I venture to hope that these discussions may assume that particular form which will prove valuable to individual physicians who are battling with disease in the smaller towns, hamlets, and remote points in the land, and who are known and properly designated as family doctors. These are the men who need your aid, who deserve the stimulus of your best thought and the inspiration of your example. As a final word, let me enter a plea in their behalf, to the end that your literature be sent to them with a liberality so characteristic of your organization, and that they be invited, when practicable, to attend your meetings.

Mr. President, it remains for me to thank you for the distinguished honor you have conferred upon me in inviting me to address this academy at this time. I appreciate it more than mere words can express, yet I regret that I have so inadequately fulfilled the responsibilities of the high office you have so kindly thrust upon me. But I assure you, sir, that I shall carry with me down to the crack of doom the memory of this pleasant visit to the New York Academy of Medicine.

284 Franklin Street.

